# Viral Capsid Is a Pathogen-Associated Molecular Pattern in Adenovirus Keratitis

**DOI:** 10.1371/journal.ppat.1000841

**Published:** 2010-04-15

**Authors:** Ashish V. Chintakuntlawar, Xiaohong Zhou, Jaya Rajaiya, James Chodosh

**Affiliations:** 1 University of Oklahoma Health Sciences Center, Department of Ophthalmology, Oklahoma City, Oklahoma, United States of America; 2 Massachusetts Eye and Ear Infirmary, Howe Laboratory, Department of Ophthalmology, Harvard Medical School, Boston, Massachusetts, United States of America; University of Michigan, United States of America

## Abstract

Human adenovirus (HAdV) infection of the human eye, in particular serotypes 8, 19 and 37, induces the formation of corneal subepithelial leukocytic infiltrates. Using a unique mouse model of adenovirus keratitis, we studied the role of various virus-associated molecular patterns in subsequent innate immune responses of resident corneal cells to HAdV-37 infection. We found that neither viral DNA, viral gene expression, or viral replication was necessary for the development of keratitis. In contrast, empty viral capsid induced keratitis and a chemokine profile similar to intact virus. Transfected viral DNA did not induce leukocyte infiltration despite CCL2 expression similar to levels in virus infected corneas. Mice without toll-like receptor 9 (Tlr9) signaling developed clinical keratitis upon HAdV-37 infection similar to wild type mice, although the absolute numbers of activated monocytes in the cornea were less in Tlr9^−/−^ mice. Virus induced leukocytic infiltrates and chemokine expression in mouse cornea could be blocked by treatment with a peptide containing arginine glycine aspartic acid (RGD). These results demonstrate that adenovirus infection of the cornea induces chemokine expression and subsequent infiltration by leukocytes principally through RGD contact between viral capsid and the host cell, possibly through direct interaction between the viral capsid penton base and host cell integrins.

## Author Summary

Adenoviruses are nonenveloped DNA viruses that infect mucosal tissues, causing a wide array of diseases. Adenovirus infection of the cornea induces inflammation in the form of multifocal leukocytic infiltrates. Although studied extensively in tissue culture models, how adenoviruses induce inflammation in the living host is not well characterized in the cornea or elsewhere. Using a unique mouse model, we studied the role of viral components in the cornea, to determine which viral part(s) induce an innate immune response. We found that neither viral DNA or viral gene expression was necessary for the development of inflammation. In contrast, viral capsid, the protein coat of the virus, induced inflammation similar to intact virus. Mice lacking the toll-like receptor 9 (Tlr9) molecule, which acts as a pathogen DNA-sensing molecule within the cell, developed clinical inflammation upon adenovirus infection similar to wild type mice. Virus associated inflammation in the mouse cornea could be blocked by treatment with a peptide containing components of the adenoviral capsid. Adenovirus infection of the cornea induces inflammation principally through contact between the viral capsid and the host cell. Our study provides new insights into how the innate immune system in the eye responds to a clinically important viral pathogen.

## Introduction

Human adenoviruses (HAdV) are major mucosal pathogens of the ocular, respiratory, and gastrointestinal tracts [1]. HAdV are also a source of disseminated infections involving multiple organs in immunocompromised patients [2], [3]. Epidemic keratoconjunctivitis (EKC) is a highly contagious infection of the eye caused principally by serotypes HAdV-8, 19, and 37. Multifocal leukocytic infiltration of the subepithelial corneal stroma is the sine qua non of EKC [4], and is associated with prolonged discomfort and poor vision. In experimental studies, infection of human keratocytes with adenoviruses results in expression of chemokines before the onset of viral gene expression [5], [6], [7], suggesting that innate immune responses can occur independently of the effects of viral gene products or viral replication. Such observations are consistent with data from studies of innate immune responses to vectors used in adenovirus based gene therapy [8], [9], [10], [11], [12], [13].

Activation of the innate immune system by microbes involves stimulation of a range of host molecular pattern recognition receptors (PRRs) that sense the unique molecular patterns present on pathogens [14]. These molecular patterns are typically distinct ligands present on the pathogens' surface or their nucleic acid. It was recently demonstrated that HAdV nucleic acids play an important role in cytokine expression after infection *in vitro* [9], [11], [15], [16]. Genomic adenoviral DNA activates multiple PRRs including Tlr9, a transmembrane protein present in the endocytic vesicles of cells that signals through the MyD88 pathway upon interaction with phosphodiester 2′ deoxyribose sugar backbone [17] or unmethylated CpG motifs of DNA [18]. Adenoviral DNA may also activate DNA-dependent activator of interferon-regulatory factors (DAI) present in the cytosol [19]. DAI is Tlr-independent [20], [21], and distinct from known sensors of double stranded RNA, retinoid-inducible gene 1 (RIG1) and melanoma differentiation-associated gene 5 (MDA5). The DAI pathway mediates type 1 interferon and chemokine expression through interferon regulatory factor 3 (IRF3), inhibitor of IκB kinase epsilon (IκBkε) and Tank binding kinase 1 (Tbk1) [19], [20], [21]. Intracytoplasmic HAdV DNA in peritoneal macrophages also induces expression of cytokines through cryopyrin/NALP3 and ASC which are components of the inflammasome [16].

Cytokine responses to adenoviral molecular patterns appear to be cytokine, cell, and molecular pattern specific. In murine peritoneal macrophages [15] and bone marrow derived macrophages [22], IL-6 expression upon adenovirus infection was mediated by Tlr9. Adenovirus infection of both human [11] and murine [9] plasmacytoid dendritic cells resulted in Tlr9-MyD88-dependent type 1 interferon expression. In murine conventional dendritic cells [9], and bone marrow macrophages [15] type 1 interferon expression induced by intact adenovirus or naked adenoviral DNA was Tlr-independent and relied on DNA sensors in the cytosol rather than in the endosome. Similarly, in adenovirus infection of murine splenic cells, type I interferon expression occurred independently of known Tlr molecules, cytosolic sensors, and IRF3, but required viral endosomal escape within the host cell [23].

Adenoviral capsid components bind to primary and secondary host cell receptors to mediate viral entry and transport. Capsid elements may also serve as virus-associated molecular patterns to activate an innate immune response. The coxsackie –adenovirus receptor (CAR) is a primary receptor used by many HAdV [24]. CAR interaction with a recombinant HAdV-5 fiber protein has been shown to activate signaling pathways *in vitro* and results in the expression of IL-6 [25]. After binding to CAR, Arg-Gly-Asp (RGD) motifs located in the penton base of adenoviruses, including HAdV-37, interact with cellular integrins, including α_v_β_1_, α_v_β_3_, α_v_β_5_, α_5_β_1_, and α_M_β_2_, leading to the internalization of HAdV via clathrin-coated pits [26], [27] and activate intracellular signaling pathways resulting in chemokine expression [8], [28]. Adenoviral empty capsids – devoid of DNA – have been shown to induce chemokine expression *in vitro* [29], [30], presumably through interactions with cellular integrins. *In vivo*, interaction of adenovirus with splenic macrophages triggered IL-1α activation in integrin (β_3_) dependent fashion [31]. However, the response to adenovirus-associated molecular patterns has not been studied in the cornea, an important site of adenovirus infection [4].

The human cornea is a specialized avascular tissue forming the outermost part of the visual axis, and is divided anatomically into epithelial, stromal, and endothelial layers. The stromal layer contains predominantly extracellular matrix, with a highly organized interconnected network of fibroblast-like cells, the keratocytes [32]. A lesser number of resident bone-marrow derived cells with dendritic cell markers and macrophages also populate the corneal stroma [33], [34], [35]. The precise arrangement of collagen fibrils and other extracellular matrix components in the corneal stroma is an important determinant of corneal transparency. [36]. Stromal cells are highly responsive to pathogenic or mechanical insult, to which they produce prodigious quantities of chemokines [37], [38]. Therefore, the corneal stroma is highly endowed with resources for innate and adaptive immune responses against ocular pathogens. Given the tissue architecture and ease of observation of the corneal stroma, the mouse cornea is an excellent model to study the interactions of specific viral molecular patterns with tissue stromal cells *in vivo*.

In this study, we show that viral capsid is a sufficient molecular pattern for the development of clinical keratitis in a mouse adenovirus keratitis model [39]. Furthermore, virus induced leukocytic infiltrates and chemokine expression in mouse cornea could be blocked by treatment with a peptide containing RGD, while viral DNA, viral gene expression, and viral replication were not essential to the development of keratitis. Viral DNA differentially stimulated IL-6 and CCL2 through Tlr9 and cytoplasmic DNA sensors, respectively, but by itself, viral DNA was insufficient to induce keratitis. Therefore, chemokine expression and cellular infiltration in adenovirus keratitis is predominantly an outcome of the interaction between viral capsid and the host cell.

## Results

### Viral Gene Expression Is Not Essential for Adenovirus Keratitis

**Figure 1:**
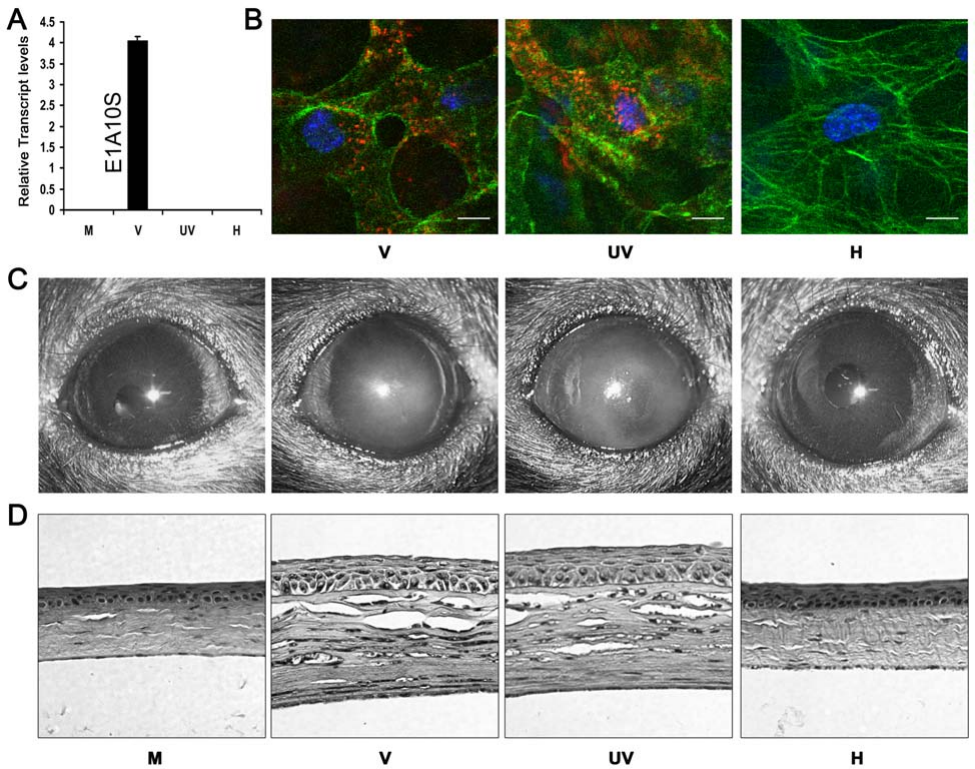
Viral gene expression is not essential for adenovirus keratitis. (A) Real-time PCR for the relative expression of viral transcript E1A10S at 4 hpi in mock (M), intact (V), UV-inactivated (UV), or heat-inactivated (H) HAdV-37 infected A549 cells. Data represents mean of three separate experiments ± SD. (B) Mouse corneas injected with Cy3-labeled intact (V), UV- inactivated (UV), or heat-inactivated (H) virus were analyzed by confocal microscopy at 90 min pi (n = 5 corneas/group). Red: Cy3-labeled virus. Green: intracellular actin (phalloidin stain). Blue: nuclei (TO-PRO3 stain). Scale bar 20 µM. (C) Representative photographs and (D) hematoxylin and eosin stained histopathological sections of mice corneas at 4 dpi, infected with virus free buffer (M), intact virus (V), UV-inactivated virus (UV), or heat-inactivated virus (H) (n = 5 mice/group).

To determine if corneal leukocytic infiltrates can be induced in the absence of viral gene expression and replication, we utilized intact, heat-inactivated, and UV-inactivated HAdV-37. Heating of the adenovirus damages its protein capsid structure, rendering it incapable of interacting with its cellular receptor, and thus from entering the host cell or triggering downstream signaling mechanisms. UV-exposure of virus damages its DNA, allowing receptor interaction, viral entry, and passage of viral DNA into the nucleus, but prevents subsequent transcription and viral replication. We tested the capacity of UV and heat treated virus to be internalized by host cells using Cy3-labeling of the virus and confocal microscopy, after validation of heat and UV-inactivation by real-time PCR for viral gene expression. When analyzed by real-time PCR, intact adenovirus showed robust transcription of its early gene E1A10S at 4 hours post-infection (hpi) in human A549 cells. In comparison, heat- and UV-inactivated adenovirus showed minimal expression at the same time-point (**Fig. 1A**). *In vivo* analysis by confocal microscopy showed that Cy3-labeled heat-inactivated virus was unable to enter corneal stromal cells at 1 hpi. In contrast, intact and UV-inactivated virus were perinuclear in location at the same time-point (**Fig. 1B**).

To determine if adenoviral gene expression was essential for the development of keratitis *in vivo*, C57BL/6J mouse corneas were injected with virus free buffer, 10^5^ TCID of intact HAdV-37, or equivalent quantities of heat- or UV-inactivated HAdV-37. Clinical examination of infected eyes showed corneal opacity developing by 1 day post-infection (dpi) in intact and UV-inactivated virus injected corneas (data not shown). This opacity peaked at 4 dpi (**Fig. 1C**). In contrast, buffer (mock infected control) and heat-inactivated virus injected corneas did not develop corneal opacity up to 4 dpi. Histopathology demonstrated corneal stromal edema and leukocyte infiltration at 4 dpi in intact and UV-inactivated virus infected corneas (**Fig. 1D**). Buffer or heat-inactivated virus injection did not cause appreciable leukocyte infiltration.

**Figure 2:**
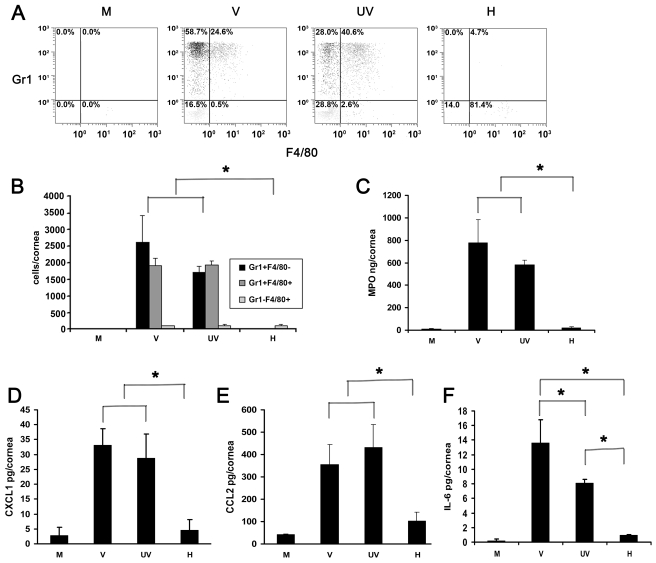
UV-inactivated adenovirus induces leukocyte infiltration and cytokine expression. (A) Representative dot plots of single cell suspensions prepared from corneas at 4 dpi stained with Gr1 and F4/80 and gated on CD45^high^ labeled cells. Corneas were infected with virus free buffer (M), or intact (V), UV-inactivated (UV), and heat-inactivated (H) HAdV-37. (B) Quantification of average numbers of Gr1 and F4/80 stained corneal cells in intact (V), UV-inactivated (UV), or heat-inactivated (H) virus injected corneas at 4 dpi (n = 6 mice/group). Data is derived from three separate experiments, and error bars represent SD. (C) Myeloperoxidase (MPO) levels assessed 24 hours post injection with virus free buffer (M), intact virus (V), UV-inactivated virus (UV), or heat-inactivated virus (H) are shown (n = 9 mice/group). Data represents mean of three independent experiments ± SD. (D–F) Cytokine expression in corneas after injection with virus free buffer (M), intact virus (V), UV-inactivated virus (UV), or heat-inactivated virus (H) as measured at 16 hpi by ELISA for CXCL1 (D), CCL2 (E), and IL-6 (F) protein (n = 9 mice/group). Data represents mean of three independent experiments ± SD. * p<.05, ANOVA.

Next, we applied flow cytometry to characterize the leukocyte phenotypes in the corneal stroma of infected animals. At 4 dpi, the numbers of infiltrating cells that were Gr1+F4/80-(polymorphonuclear neutrophils) and Gr1+F4/80+ (inflammatory monocytes) [40] were similar (*p*>.05) in intact virus and UV-inactivated virus injected corneas. However, both groups had significantly higher number of Gr1+F4/80− and Gr1+F4/80+ cells when compared to corneas injected with either buffer or heat-inactivated virus (*p*<.05) (**Fig. 2A and B**). The phenotypes and proportion of the leukocytes after injection of intact virus and UV-inactivated virus did not differ. Levels of myeloperoxidase (MPO), a surrogate indicator for the extent of neutrophil infiltration, were also significantly higher at 24 hpi in intact and UV-inactivated virus injected corneas than after injection of buffer or heat-inactivated virus (**Fig. 2C**).

CXCL1 and CCL2 have been shown to be expressed in adenovirus infection and are paradigm chemokines responsible for neutrophil and monocyte chemotaxis, respectively [41], [42]. IL-6 was shown to be expressed early in the mouse model of adenoviral pneumonia [43]. We next tested for the expression of CXCL1, CCL2 and Interleukin-6 (IL-6) at 16 hpi. Levels of all the three cytokines were elevated in intact virus and UV-inactivated virus injected corneas as compared to the values from buffer- or heat-inactivated virus injected corneas (*p*<.05) (**Fig. 2D–F**). CXCL1 and CCL2 expression after infection with UV-inactivated virus was not statistically different from that with intact virus. However, intact virus induced IL-6 expression to a greater degree than UV-inactivated virus (p<.05) (**Fig. 2F**).

### Tlr9 Is Not Essential for Innate Immune Responses in Adenovirus Keratitis

**Figure 3:**
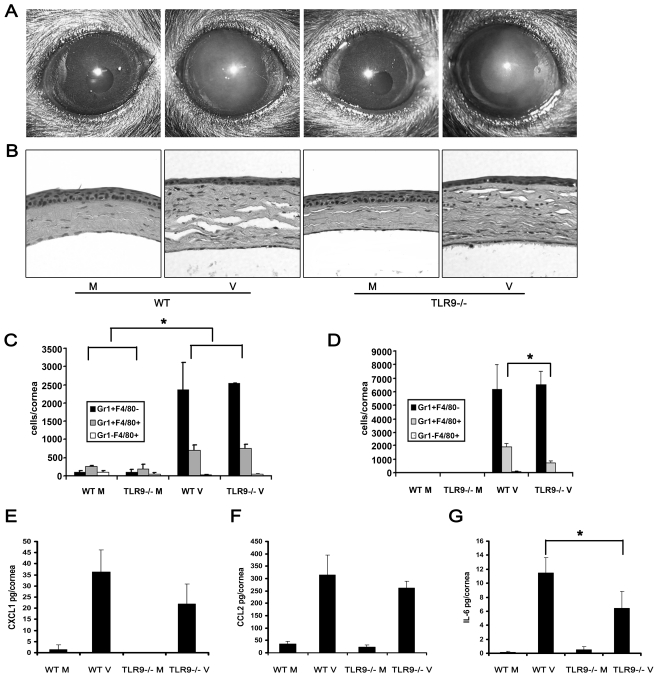
Tlr9 is not essential for the development of adenovirus keratitis. (A and B) Wild type and Tlr9^−/−^ corneas were infected with virus free buffer (M) or HAdV-37 (V) and observed for 4 dpi. Representative photographs (A) and histopathology sections (B) of corneas at 4 dpi are shown (n = 5 mice/group). (C and D) Flow cytometric analysis for Gr1 and F4/80 positive cells in corneas of wild type and Tlr9^−/−^ mice after injection with virus free buffer (M) or virus (V) at 1 (C) and 4 (D) dpi is shown (n = 6 corneas/group). Data represents mean of three independent experiments ± SD. (E–G) Protein levels of CXCL1 (E), CCL2 (F) and IL-6 (G) were assayed by ELISA at 16 hpi in wild type and Tlr9^−/−^ mice corneas injected with virus free buffer (M) or virus (V) (n = 9 corneas/group). Data is mean of three separate experiments, and error bars denote SD. * p<.05, ANOVA.

Tlr9 is a pathogen-associated molecular pattern receptor present in intracellular endocytic vesicles, and is activated by the presence of unmethylated CpG motifs and the phosphodiester sugar DNA backbone [17], [18]. Tlr9 is the critical toll-like receptor for cellular recognition of nucleic acid in DNA viruses [44], [45], [46]. Tlr9 is also expressed in the murine cornea and has been implicated in the pathogenesis of experimental viral and bacterial keratitis [47], [48]. Because UV-inactivated virus induced keratitis to a similar degree as that induced by intact virus (**Figs. 1** **and** **2**), the possibility remained that viral CpG motifs and DNA in UV-inactivated virus might be activating Tlr9 in corneal cells. To test this possibility, we infected wild type and Tlr9^−/−^ corneas with HAdV-37. Development and progression of corneal opacities in wild type and Tlr9^−/−^ mice appeared similar at 1 (data not shown) and at 4 dpi (**Fig. 3A**). Buffer injection did not result in corneal opacity in either mouse group. Histology of virus infected corneas at 4 dpi demonstrated a similar pattern of stromal infiltration by leukocytes in wild type and Tlr9^−/−^ mice (**Fig. 3B**). By flow cytometry at 1 dpi, both Tlr9^−/−^ and wild type mice infected with virus showed a similar degree of infiltration with Gr1+F4/80− and Gr1+F4/80+ cells (p>.05) (**Fig. 3C**). However, at 4 dpi, the Gr1+F4/80+ cells were significantly less in Tlr9^−/−^ mice as compared to wild type mice (p<.05) (**Fig. 3D**), suggesting that Tlr9 might play a role in the sustained infiltration of monocytes into the adenovirus infected cornea. The levels of CXCL1 and CCL2 protein also appeared comparable in wild type and Tlr9^−/−^ corneas infected with adenovirus (**Fig. 3E and F**). However, IL-6 levels were significantly less in Tlr9^−/−^ corneas (p<.05) (**Fig. 3G**), suggesting a role for Tlr9 in IL-6 induction by adenovirus, as seen previously [22]. Buffer injection did not cause significant upregulation of cytokines in either group of mice, and viral replication was absent in Tlr9^−/−^ corneas (data not shown), as was previously shown in corneas of wild type mice [39].

### Adenoviral Genomic DNA Does Not Induce Keratitis

**Figure 4:**
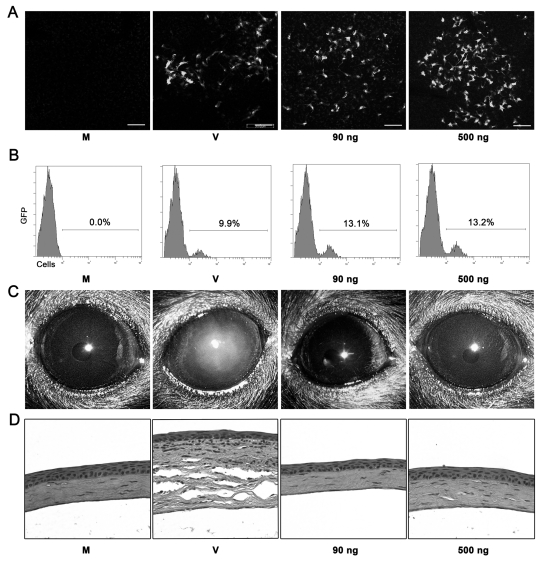
Adenoviral genomic DNA is not sufficient to induce keratitis in mice. (A) Confocal microscopy of mouse corneal stroma at 1 day after mock treatment with transfection reagent alone (M), HAdV-5 vector expressing eGFP (V), or plasmid vector EGFP-C1 (90 or 500 ng DNA). Photographs are representative of three corneas in each group. Scale bar, 200 µM. (B) Flow cytometric analysis of corneas at 1 day after injection with transfection reagent alone (M), plasmid vector (90 or 500 ng DNA) and transfection reagent, or HAdV-5 (V) vector expressing eGFP. Numbers in histograms denote percentage of total cells expressing eGFP. (C and D) C57BL/6J mouse corneas were injected with virus free buffer (M), HAdV-37 (V) or 90 ng and 500 ng of HAdV-37 genomic DNA with transfection reagent and observed up to 4 dpi. Representative photographs (C) and histopathology sections (D) of corneas at 4 dpi are shown (n = 5 mice/group).

Recently, it was demonstrated that DNA in the cytoplasm of mammalian cells can initiate Tlr-independent innate immune responses [16], [20], [21]. In light of our results in Tlr9^−/−^ mice, we sought to test the hypothesis that viral DNA might be stimulating inflammation by another pathway. First, to confirm our ability to deliver DNA to corneal cells, we injected enhanced green fluorescent protein (eGFP) expressing plasmid vector into mouse corneas. Robust expression of eGFP was seen at 1 dpi and was similar to expression of eGFP by a HAdV-5 vector (**Fig. 4A**). At 1 dpi, transfection efficiency of plasmid, as measured by flow cytometry, was comparable to that of HAdV-5 vector (**Fig. 4B**). To analyze the development of keratitis after transfection of adenoviral DNA, corneas were transfected with Ava1 digested HAdV-37 genomic DNA in two different concentrations, the first equal to the amount of DNA contained in 10^5^ TCID of intact HAdV-37 (90 ng), and the second, a roughly 5 ½ -fold higher concentration (500 ng). This enzyme was chosen because it digests the DNA into small enough fragments to be efficiently taken up by transfected cells, with resulting fragments still of sufficient size to activate cytosolic DNA sensors [20].Transfection of viral DNA did not induce corneal opacity up to 4 dpi (**Fig. 4C**). Histopathology also did not show appreciable leukocytic infiltration in DNA transfected corneas at 4 dpi (**Fig. 4D**).

**Figure 5:**
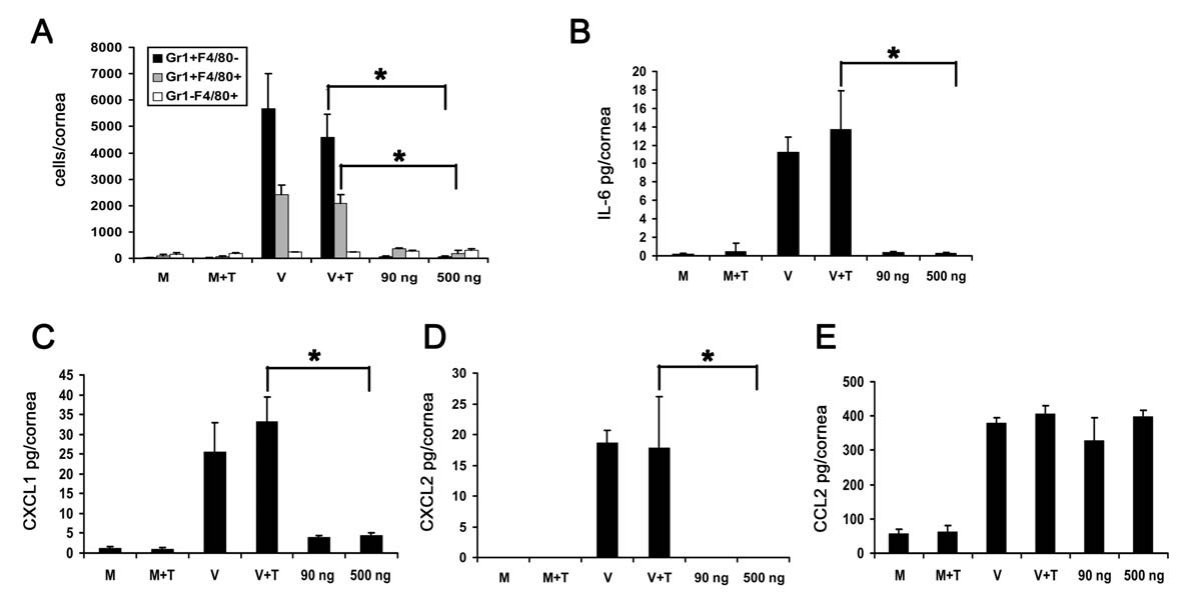
Adenoviral genomic DNA induces differential expression of cytokines but does not cause infiltration of leukocytes into the cornea. (A) Flow cytometric analysis of Gr1 and F4/80 positive cells in mouse corneas at 4 days after injection with virus free buffer (M), virus free buffer and transfection reagent (M+T), intact HAdV-37 (V), intact HAdV-37 and transfection reagent (V+T), and 90 ng or 500 ng of HAdV-37 genomic DNA with transfection reagent (n = 6 corneas/group). Data shown represents the mean of three independent experiments, and error bars represent SD. (B–E) Protein levels of cytokines IL-6 (B), CXCL1 (C), CXCL2 (D) and CCL2 (E) in mouse corneas at 16 hpi. Corneas were injected with virus free buffer (M), virus free buffer and transfection reagent (M+T), intact HAdV-37 (V), intact HAdV-37 and transfection reagent (V+T), and 90 ng or 500 ng of HAdV-37 genomic DNA with transfection reagent (n = 9 corneas/group). Data shown represents the mean of three separate experiments, and error bars denote SD. * p<.05, ANOVA.

In addition, we analyzed the response to DNA transfection by flow cytometry. Infiltrating Gr1+F4/80− and Gr1+F4/80+ cells were significantly higher in intact HAdV-37 infected corneas when compared to DNA injected and mock injected corneas (**Fig. 5A**). As previously mentioned, naked DNA has been shown to initiate expression of chemokines and cytokines [16], [20], [21]. Intact virus induced significant upregulation of IL-6, CXCL1, and CXCL2 at 16 hpi when compared to transfected DNA (p<.05) (**Fig. 5B–D**, respectively). In contrast, CCL2 levels were significantly higher in both intact virus and transfected DNA treated corneas as compared to mock infection (**Fig. 5E**). These data suggest that CCL2 expression in adenovirus infection may be dependent upon the presence of viral DNA, but independent of Tlr9.

### Adenoviral Capsid Is Sufficient for the Development of Adenovirus Keratitis

**Figure 6:**
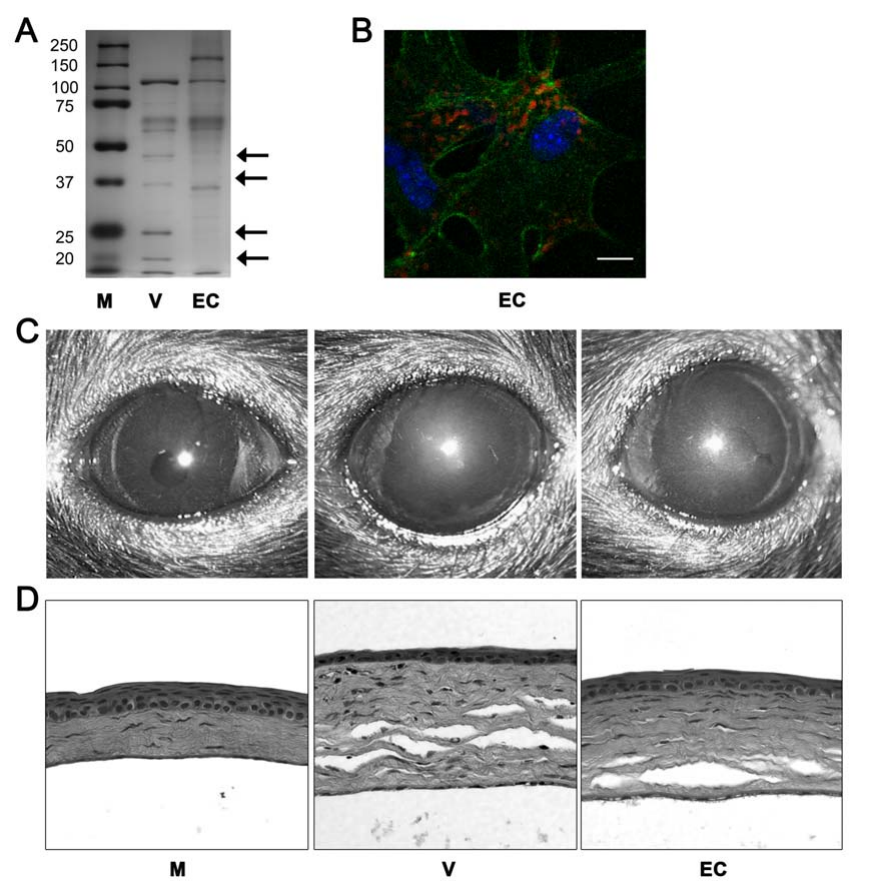
Empty adenoviral capsid is sufficient to induce keratitis. (A) Silver stained polyacrylamide gel of proteins from intact HAdV-37 (V) or empty capsid (EC). First lane (M) shows protein standards. Arrows on the right point to capsid proteins missing from the empty capsid; capsid proteins V and VII are marked by the second and fourth arrows from the top, respectively. (B) Mouse cornea injected with Cy3 dye-labeled empty capsid (EC). Intracellular virus position was visualized with confocal microscopy at 90 min pi (n = 3 corneas). Red: Cy3-labeled empty capsid. Green: intracellular actin (phalloidin stain). Blue: nuclei (TO-PRO3 stain). Scale bar 20 µM. (C) Clinical appearance and (D) histopathology of mouse corneas at 4 dpi. Corneas were injected with virus free buffer (M), intact virus (V), or empty capsid (EC) (n = 5 mice/group).

Human adenovirus has been shown to induce expression of CXCL8 within minutes of infection in human cells via activation of intracellular signaling [5], [49], [50], suggesting that interactions between viral capsid and host cellular receptor(s) may be mediating cell signaling and the downstream expression of chemokines. However, the ability of empty capsids to mediate chemokine expression has not been tested *in vivo*. We confirmed the purity of our empty capsid preparation by silver staining, which showed the absence of adenoviral core proteins V or VII in empty capsid preparations after polyacrylamide gel electrophoresis (**Fig. 6A**), and by real-time PCR, which showed no genomic DNA in empty capsid (data not shown). We further confirmed the competence of our empty capsid preparations by the entry of Cy3-labeled capsid into human corneal fibroblasts *in vitro* at 1 hpi (**Fig. 6B**). Endotoxin was not detectable in our empty capsid preparations (data not shown) and cannot be responsible for the observed inflammatory response to empty capsid *in vivo*.

Empty capsid at a protein concentration similar to 10^5^ TCID of intact HAdV-37 did not induce any visible corneal opacity up to 4 dpi (data not shown). Because empty HAdV capsids are known to be structurally unstable and therefore may be less robust ligands [29], we also tested a more concentrated capsid preparation. When empty capsid was concentrated 5-fold and injected, corneal opacities developed by 4 dpi in all mice (**Fig. 6C**). Similarly, histopathological examination at 4 dpi showed infiltration of leukocytes and formation of characteristic subepithelial infiltrates in the corneal stroma of mice injected with concentrated empty capsid (**Fig. 6D**).

**Figure 7:**
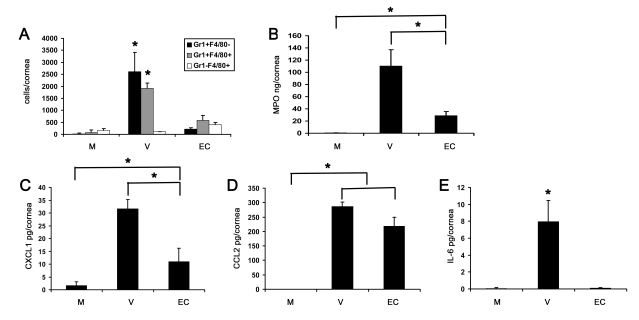
Empty viral capsid induces chemokine expression and infiltration of leukocytes into the cornea. (A) Infiltrating leukocytes were quantified using flow cytometry in corneas 4 days after injection with virus free buffer (M), intact HAdV-37 (V), or empty viral capsid (EC) (n = 6 corneas/group). Data represents the mean of three separate experiments, and error bars denote SD. (B) Myeloperoxidase (MPO) levels were quantified in mouse corneas 2 days after injection with virus free buffer (M), intact virus (V), or empty capsid (EC) (n = 6 corneas/group). Data represents the mean of two separate experiments, and error bars denote SD. (C–E) Cytokine protein levels as measured by ELISA in corneas 16 hours after injection with virus free buffer (M), intact virus (V), or empty capsid (EC). CXCL1 (C), CCL2 (D), and IL-6 (E) protein levels are shown (n = 9 corneas/group). Data shown represents the mean of three independent experiments, and error bars represent SD. * p<.05, ANOVA.

Flow cytometry at 4 dpi demonstrated lower levels of Gr1+F4/80− and Gr1+F4/80+ cells after concentrated empty capsid injection than with intact virus (p<.05) (**Fig. 7A**). However, leukocyte infiltration and MPO levels were significantly higher in concentrated empty capsid infection as compared to mock injected corneas (p<.05) (**Fig. 7A and B**, respectively). Empty capsid injections also induced expression of CXCL1 and CCL2, but not IL-6 (**Fig. 7C–E**, respectively), suggesting differential regulation of these cytokines by capsid proteins.

### Competitive Inhibition of Adenovirus Keratitis with RGD Containing Peptide

**Figure 8:**
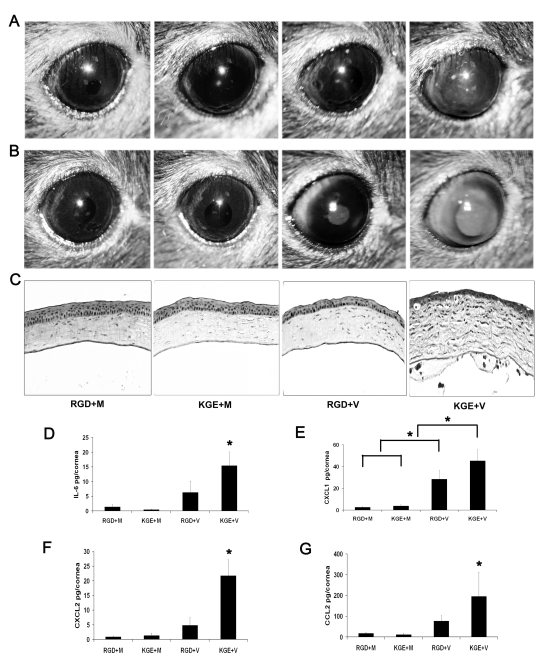
RGD inhibits leukocyte infiltration and cytokine expression in the adenovirus infected cornea. (A and B) Mouse corneas injected with RGD and virus free buffer (RGD+M), KGE and virus free buffer (KGE+M), RGD and intact virus (RGD+V), or KGE and intact virus (KGE+V) were investigated for clinical appearance at 1 and 4 dpi, respectively (n = 8 corneas/group). (C) Hematoxylin and eosin stained histopathological sections (bottom row) of mice corneas at 4 dpi. (D–G) Protein levels of cytokines IL-6, CXCL1, CXCL2 and CCL2 in mouse corneas at 16 hpi (n = 8 corneas/group). Data shown represents the mean of four separate experiments, and error bars denote SD. * p<.05, ANOVA.

Most HAdV use integrins α_v_β_3_ or α_v_β_5_ as an entry receptor through interaction with an Arg-Gly-Asp (RGD) sequence in the virion penton base protein [26]. β_3_ integrin was recently shown critical to IL-1 signaling in the adenovirus infected mouse spleen [31]. We next utilized a 15-mer synthetic peptide encompassing an RGD motif and a control peptide with RGD replaced with the amino acids Lys-Gly-Glu (KGE) to study the role of corneal integrins in viral capsid induced inflammation. We first confirmed that the RGD-containing peptide prevented corneal cell adhesion to plastic tissue culture plates; adhesion was also restricted by EDTA (data not shown). The KGE-containing peptide exerted no effect on cell adhesion. We co-injected RGD or KGE containing peptides with HAdV-37 or virus free buffer into the corneas of wild-type mice. Clinical examination of infected eyes showed corneal opacity at 1 dpi in KGE plus virus (KGE+V) injected corneas (**Fig. 8A**), peaking at 4 dpi (**Fig. 8B**). In contrast, neither RGD plus buffer (RGD+M), KGE plus buffer (KGE+M), or RGD plus virus (RGD+V) injected corneas developed corneal opacities (**Fig. 8B**). Histopathology demonstrated corneal stromal edema and leukocyte infiltration with formation of subepithelial infiltrates at 4 dpi only in KGE+V infected corneas (**Fig. 8C**). When co-injected with virus, RGD, but not KGE, also decreased expression of IL-6, CXCL1, CXCL2 and CCL2 (p<.05) (**Fig. 8D–G**, respectively) although RGD+V injected corneas also had more CXCL1 than controls (p<.05) (**Fig. 8E**), suggesting that RGD inhibition of cytokine induction was incomplete. These data suggest that RGD blocks a critical step in adenovirus induced chemokine expression and subsequent leukocyte infiltration.

## Discussion

Pathogen-associated molecular patterns (PAMP) are unique molecular ligands on or within microbes that induce activation of innate immunity through specific receptors on or within target cells. Except for a recent manuscript demonstrating the importance of the RGD-β_3_ integrin interaction in splenic macrophages [31], and a previous study demonstrating reduced innate immune responses in NALP3−/−mice [16], little is known about PAMPs in adenovirus infections *in vivo*. The purpose of our study was to elucidate adenovirus-associated molecular patterns and their specific role in innate immune responses in a living animal, using a defined disease model [39], [51].

Liu and Muruve [28] previously showed that adenoviral vectors activate innate immune responses in the liver independently of viral gene expression or viral replication. Similarly, in our keratitis model, we demonstrate that a UV-inactivated (transcriptionally inactive) adenovirus can initiate an innate immune response in the cornea. The degree of corneal opacity, cytokine expression, and cellular infiltration was similar to that induced by intact virus. These data are consistent with a murine model of adenoviral pneumonia, in which viral replication was not essential for pneumonitis [43], and suggest that activation of specific innate immune responses by adenovirus do not require viral gene expression or viral replication.

Several recent studies have shown the importance of viral CpG motifs and viral DNA in the initiation of innate immune responses against adenovirus infection *in vitro*. Type 1 interferon expression upon adenovirus infection of plasmacytoid dendritic cells was dependent upon the Tlr9 pathway [9], [11]. In contrast, conventional dendritic cells were activated by adenoviruses independent of Tlr signaling [9]. Blocking Tlr9 attenuated innate immune responses after intravenous administration of helper dependent adenovirus vectors in mice [22]. In our studies, we demonstrated keratitis in Tlr9^−/−^ mice similar to that in wild type mice. Furthermore, expression of CXCL1 and CCL2 was comparable in Tlr9^−/−^ and wild type mice. However, we did show significantly less IL-6 expression in Tlr9^−/−^ as compared to wild type mice. Similarly, expression of IL-6 after intravenous administration of adenovirus or adenovirus infection of bone marrow macrophages was dependent on Tlr9 and Myd88, respectively [15], [22]. In our model, the infiltration of Gr1+F4/80+ inflammatory monocytes at 4 dpi was also reduced in Tlr9^−/−^ mice compared to wild type. These data suggest that viral genomic DNA may differentially simulate cytokine expression and play a role in mononuclear cell infiltration, but is not essential to the development of keratitis. The murine corneal stroma contains cells of various lineages, including macrophages and bone marrow derived antigen presenting cells, in addition to the fibroblast-like keratocytes [33], [34]. Whether different resident corneal cells disparately produce cytokines in response to Tlr9 activation is not presently known. Plasmacytoid dendritic cells have not been demonstrated to date in the murine or human cornea, and in preliminary experiments, we were unable to show interaction of HAdV-37 with corneal stromal macrophages (Zhu and coworkers, unpublished data).

Cytoplasmic sensors of DNA have also been implicated in innate immune responses to DNA transfection of mammalian cells [16], [19], [20], [21]. Hence, we wished to investigate the role of viral genomic DNA in adenoviral keratitis. We injected adenoviral genomic DNA in an amount equivalent to, or 5-fold greater than, that contained in 10^5^ TCID of intact virus. Injection of either of these concentrations failed to induce clinical keratitis, but did induce expression of CCL2 and a modest mononuclear cell infiltrate. Similarly, adenoviral DNA transfected in both macrophages and lung fibroblasts induced expression of CCL2 via an interferon regulatory factor 3 mediated pathway [15]. These results again suggest differential control of cytokine expression by different PAMP receptors, possibly due to the diversity of cell types within the corneal stroma. The lack of keratitis upon viral DNA transfection was not due to differences in uptake between intact virus and naked DNA, as the efficiency of transfection and transduction was equivalent. Taken together, the experiments with Tlr9^−/−^ mice and DNA transfections confirm that viral genomic DNA contributes to cytokine expression and infiltration of mononuclear cells, but is not sufficient to induce clinical keratitis.

Because viral DNA and CpG motifs were not sufficient for the development of keratitis, we next examined viral capsid as a PAMP. We prepared HAdV-37 empty capsid, lacking the central nucleoprotein core of intact virus. Adenoviral empty capsid has been shown to be somewhat unstable [29], and free fibers in the preparation may prevent capsid – receptor interactions and reduce binding and downstream signaling [52]. Despite these limitations, empty capsid induced clinical keratitis, chemokine expression, and infiltration of both neutrophils and monocytes. These data suggest that viral capsid is a major virus-associated molecular pattern for adenovirus keratitis. Interestingly, IL-6 expression was not induced by empty viral capsid, but was dependent on Tlr9. In addition, the clinical keratitis and leukocyte infiltration due to empty capsid were less than that induced by intact virus. Reduced innate immune responses to capsid might be due to the unstable structure of empty viral particles and a less efficient interaction with host cell viral receptors [29], [52]. Alternatively, the complete innate immune response to adenovirus in the cornea might require the combined effects of both viral capsid and viral DNA. We further showed that a peptide containing RGD, as in the HAdV-37 penton base [53] inhibited leukocyte infiltration associated with virus infection, whereas an otherwise identical molecule except for the RGD did not. Treatment with the RGD containing peptide also deceased cytokine expression in adenovirus infected corneas. Taken together, these data indicate that RGD sequence within the adenovirus penton base is critically important not only for internalization [54], but also for inflammation, possibly through interaction with β_3_ integrin [31].

The mechanism of viral capsid interaction with the target host cell varies greatly between different tissue and cell types. For example, adenovirus vectors have been shown to bind neutrophils via Fc receptors and complement receptor 1 [55]. The transduction of liver cells by intravenously administered adenovirus vectors was facilitated by clotting factors [12], [13]. In other and diverse cell types, adenovirus infection proceeds via an integrin-dependent mechanism [54]. Stromal cells in the cornea express α_v_ and β_3_ integrins (unpublished data, Chintakuntlawar and Chodosh). In human corneal epithelial cell culture, growth of cells on vitronectin, a ligand for α_v_β_3_, enhanced replication of HAdV-19 [56]. Our laboratory also previously demonstrated in human corneal fibroblasts infected with HAdV-19, that CCL2 and CXCL8 expression was mediated by intracellular signaling activated by viral binding [5], [6]. However, the exact nature of adenovirus interaction with corneal stromal cells *in vivo* is unknown and remains to be studied.

It is important to determine the primary initiating event in the innate immune response to any pathogen. In this study, we show that viral capsid is an essential PAMP for the induction of adenovirus keratitis in the mouse model. Keratitis was not dependent upon viral gene expression, viral replication, or the presence of viral DNA. Further studies will be necessary to delineate the cell types responsible for specific responses to adenovirus in the cornea.

## Materials and Methods

### Ethics Statement

All animals were treated according to the Association for Research in Vision and Ophthalmology (ARVO) statement for the use of animals in ophthalmic and vision research and all experimental protocols were approved by the Institutional Animal Care and Use Committee at the University of Oklahoma Health Sciences Center, and the Animal Care Committee of the Massachusetts Eye and Ear Infirmary.

### Cells, Virus and Animals

Eight to 12-week-old wild type female C57BL/6J mice were purchased from Jackson Laboratories (Bar Harbor, ME). Tlr9^−/−^ mice on C57BL/6J background were kind gift from Dr. Paul Kincade (Oklahoma Medical research Foundation, Oklahoma City) and Dr. Shizuo Akira (Osaka University, Osaka, Japan). Human lung carcinoma cell line A549 was obtained from American Type Culture Collection (ATCC, Manassas, VA). Cells were maintained in Dulbecco's modified Eagle's medium containing 10% heat-inactivated fetal bovine serum.

Human adenovirus 37 (HAdV-37) was obtained from ATCC and purified by cesium chloride gradient. UV-inactivation of the virus was done by irradiating the virus on ice with UV light of wavelength 254 nm at a distance of 15 cm for 20 minutes. Heat-inactivation of the virus was done by incubating in a water bath at 56°C for 30 minutes. Empty capsids were prepared by harvesting the upper band in cesium chloride gradient purification, followed by overnight centrifugation at 38000× *g* on a continuous cesium chloride gradient followed by overnight dialysis against the dialysis buffer (10 mM Tris, 80mM NaCl, 2mM MgCl_2_, and 10% glycerol). Empty capsids were concentrated five-fold using centrifugal filter units (Millipore, Billerica, MA) and concentrations were measured by bicinchoninic acid protein assay (Pierce, Rockford, IL). Virus was titered in triplicate using A549 cells.

### Experimental Infections

Mice were anesthetized by intramuscular injection of ketamine (85 mg/kg) and xylazine (14 mg/kg). Anesthetic drops (0.5% proparacaine hydrochloride, Alcon, Fort Worth, TX) were applied topically to each eye before injections. One microliter of virus free dialysis buffer, HAdV-37 (10^5^ TCID [tissue culture infective dose]), UV-inactivated HAdV-37, heat-inactivated HAdV-37, empty HAdV-37 capsid or Ava1 digested HAdV-37 DNA was injected in the center of corneal stroma with a glass micropipette needle fitted with a gas-powered microinjection system (MDI, South Plainfield, NJ) under an ophthalmic surgical microscope (Carl Zeiss Meditec, Inc., Thornwood, NY). At indicated time-points after injection, mice were euthanatized using CO_2_ inhalation and corneas were dissected and processed for further analysis.

### Inhibition of Infection by Adenoviral Penton Base Peptides

Synthetic 15-mer peptides were obtained from GenScript Corporation (Piscataway, NJ) and were reported to be >90% pure by the manufacturer. The sequence of wild type penton base peptide including RGD was PPKR**RGD**LAVLFAKV, and the negative control peptide, which had KGE in place of RGD, was PPKR**KGE**LAVLFAKV. The peptides were dissolved in water, diluted in phosphate-buffered saline (PBS), and 0.5 µl RGD (2 mM) or KGE (2 mM) containing peptide was mixed with 0.5 µl HAdV-37 (2×10^5^ TCID) or virus free dialysis buffer and injected in the corneal stroma of wild type mice as described above. Mouse corneas were removed at indicated time-points.

### PCR for Viral Gene Transcription

Intact, UV-inactivated and heat-inactivated HAdV-37 were used to infect human A549 cells. Four hpi total RNA was isolated by single step isolation method using TRIzol (Invitrogen, Eugene, OR) according to the manufacturer's instructions. Following DNase treatment (Ambion, Austin, TX), 2 µg of total RNA was used to synthesize cDNA using reverse transcriptase (Superscript II , Invitrogen). A total of 2 µL of cDNA obtained by reverse transcription was used for amplification in a final volume of 20 µL containing 10 µL of 2× SYBR green master mixes (Applied Biosystems [ABI], Foster City, CA) and 250 nM of specific forward and reverse primers. RNA concentrations of samples were normalized using quantification of GAPDH mRNA as the internal control. E1A10S primers were as follows, forward 5′ GGAGGTAGATGCCCATGATGA 3′ and reverse 5′ GTTGGCTATGTCAGCCTGAAGA 3′. GAPDH primers were as follows, forward 5′ GACAATGAATACGGCTACAGCAACAGG 3′ and reverse 5′ GTTGGGATAGGGCCTCTCTTGCTCA 3′. Quantitative real-time PCR amplification and analysis was performed as described previously [39].

### ELISA

Mouse corneas were removed at indicated time-points (n = 3/time-point/group) and flash frozen in liquid nitrogen. Corneas were then homogenized in 400 µL of PBS with 1 mM phenylmethylsulfonyl fluoride (PMSF), 1 µg/mL aprotinin, and 10 µg/mL leupeptin (Sigma-Aldrich, St. Louis, MO). The lysates were centrifuged at 10,000× *g* for 10 minutes at 4°C, and the supernatants were used for ELISA. Mouse CXCL1 (KC), CXCL2 (MIP-2), CCL2 (MCP-1), IL-6 (all from R&D Systems, Minneapolis, MN) and myeloperoxidase (Cell Sciences, Canton, OH) protein detection was performed with commercially available sandwich ELISA kits with capture and detection antibodies, according to the manufacturer's instructions. Each sample and standard was analyzed in duplicate. The plates were read on a microplate reader (Molecular Devices, Sunnyvale, CA) and analyzed (SOFTmax software; Molecular Devices).

### Histopathology and Immunohistochemistry

Injected mouse corneas were removed, rinsed in PBS, and fixed with 10% neutral buffered formalin for 24 hours at room temperature. After paraffin embedding, whole eyes were cut into 5-µm-thick sections, mounted on positively charged slides and air dried overnight. After deparaffinization and rehydration, slides were stained with hematoxylin and eosin. Slides were coverslipped using a synthetic resin, and photographed (Axiovert 135; Carl Zeiss Meditec, Inc.), using a 40× objective.

### Cy3-Labeling of Virus and Confocal Microscopy

Intact HAdV-37, and empty capsids were conjugated with Cy3 dye (GE Healthcare, Piscataway, NJ) as per Leopold and co-workers [57]. One milligram of Cy3 dye was reconstituted in 1 mL of 0.1 M sodium bicarbonate (pH 9.3). Labeling was performed by conjugating Cy3 dye to virus at a concentration approximately equal to 10^12^ Ad particles/mL, where reconstituted Cy3 dye was 20% of the final solution. The mixture was allowed to incubate for 30 minutes in the dark with gentle mixing every 10 minutes, followed by overnight dialysis to remove the excess Cy3 dye.

### *In Vivo* DNA Transfection

Adenoviral DNA was isolated from purified HAdV-37 by phenol chloroform extraction. Adenoviral DNA was further digested with restriction enzyme Ava1 (Promega, Madison, WI) and purified by phenol chloroform extraction, followed by ethanol precipitation. After careful washing, DNA was suspended in nuclease-free water and stored at −20°C. *In vivo* transfection of the mouse corneal stroma was done according to the method described by Mohan et al. [58]. DNA was mixed with 100 nm of DOPE (dioleoyl phosphatidyl ethanol amine) and 100 nm of DDAB (dimethyl dioctadecyl ammonium bromide) cationic lipids in phosphate buffered saline and incubated on ice for 1 hour before injections. One microliter of mixture containing 90 or 500 ng of adenoviral genomic DNA or transfection reagent alone was injected in the mouse cornea. Mice were euthanized at indicated time-points and corneas dissected for further analysis. Similarly, to measure the transfection efficiency of DNA injection, a plasmid vector (EGFP-C1, Clontech, Mountain View, CA) or HAdV-5 vector expressing eGFP were injected as described above. The mice corneas were dissected at 1 day post-injection for confocal microscopy and flow cytometry.

### Confocal Microscopy

Cells and whole corneas (cut radially to flatten them) were fixed with 4% paraformaldehyde for 30 minutes at 25°C, and coverslipped using mounting medium containing DAPI (4,6-diamidino-2-phenylindole; Vectashield; Vector Laboratories, Burlingame, CA). Samples were scanned with confocal laser scanning microscope (IX81-FV500; Olympus, Melville, NY). Whole corneas were scanned in the *z*-axis with a step size of 1–2 µm. The microscope system software (FluoView; Olympus) was used for analysis.

### Flow Cytometry

Corneas were dissected from mouse eyes at the indicated time-points following infection. The corneas were cut into small (1–2 mm segments) pieces and digested with 1 mg/ml collagenase type I (Sigma Chemical Co., St. Louis, MO) for 2 hours triturating the sample every 15 minutes. Single cell suspensions were washed twice (300× g, 5 min/wash) in PBS and then incubated on ice for 15 min with 2 µl anti-mouse Fc block (BD Pharmingen, San Diego, CA) in a total volume of 100 µl PBS-1% BSA. Following the incubation, the cells were centrifuged (300× g, 5 min) and resuspended in 5% normal rat serum (Jackson Immuno Research Inc., West Grove, PA) for an additional 15 min on ice. Cells were then triple labeled with 6 µl containing 2 µl FITC-conjugated anti-mouse F480 (CI:A-3), 2 µl phycoerythrin-Cy5-conjugated anti-CD45 (clone 30-F11), and 2 µl PE-conjugated anti-mouse Gr1 (RB68C5) and incubated in the dark on ice for 30 min. Following the incubation period, the cells were washed 3 times with PBS-1% BSA (300× g, 5 min/wash) and resuspended in PBS containing 1% paraformaldehyde. After overnight fixation at 4°C in the dark, cells were pelleted (300× g, 5 min/wash) and resuspended in PBS-1% BSA. Immediately before analysis, CountBright absolute counting beads (Invitrogen) were added (21600 beads/sample). Cell suspensions were gated on CD45^high^ labeled cells, and the percentage of each cell type were determined at this gate setting. A second gate was established to count the number of beads that passed through during the run (300 sec). The absolute number of cells per cornea were determined by calculating the number of beads counted in 300 seconds/21600× number of cells in the CD45^high^-gated sample.

For *in vivo* DNA transfection efficiency experiments corneas were dissected at 1 day pi and incubated in 10 mM EDTA in phosphate buffered saline for 30 minutes at 37°C. Epithelial sheet was stripped from underlying stroma using smooth forceps. Corneal stroma was digested and washed as described above and stained with propidium iodide to exclude dead cells. GFP positive cells were counted as percentage of total cells to measure the efficiency of transfection.

### Statistical Analysis

Real-time PCR, ELISA and flow cytometry experiments were each performed at least three times. Mean of observations from three experiments were compared by ANOVA with the Scheffé multiple comparison test using statistical analysis software (SAS institute Inc. Cary, NC). Statistical significance was set at α = .05.

### List of Accession/ID Numbers for Genes and Proteins

1. Human Adenovirus-37: ACCESSION NO. DQ900900
2. Human Adenovirus-5: ACCESSION NO. AC_000008
3. E1A10S: ACCESSION NO. DQ900900
4. Toll-Like Receptor-9 (Mus musculus): GENE ID. 81897
5. Interleukin-6 (Mus musculus): GENE ID. 16193
6. CCL-2 (Mus musculus): GENE ID. 20296
7. CXCL-1 (Mus musculus): GENE ID. 14825
8. CXCL-2 (Mus musculus): GENE ID. 20310
9. Myeloperoxidase: GENE ID. 17523
